# A case for genomic medicine in South African paediatric patients with neuromuscular disease

**DOI:** 10.3389/fped.2022.1033299

**Published:** 2022-11-17

**Authors:** Sharika V. Raga, Jo Madeleine Wilmshurst, Izelle Smuts, Surita Meldau, Soraya Bardien, Maryke Schoonen, Francois Hendrikus van der Westhuizen

**Affiliations:** ^1^ Department of Neurophysiology, Department of Paediatric Neurology, Red Cross War Memorial Children's Hospital, Neuroscience Institute, University of Cape Town, Cape Town, South Africa; ^2^ Department of Paediatrics, Steve Biko Academic Hospital, University of Pretoria, Pretoria, South Africa; ^3^ Division of Chemical Pathology, Department of Pathology, National Health Laboratory Service and University of Cape Town, Cape Town, South Africa; ^4^ Division of Molecular Biology and Human Genetics, Faculty of Medicine and Health Sciences, Stellenbosch University, Cape Town, South Africa; ^5^ South African Medical Research Council/Stellenbosch University Genomics of Brain Disorders Research Unit, Cape Town, South Africa; ^6^ Human Metabolomics, North-West University, Potchefstroom, South Africa

**Keywords:** Africa, neuromuscular disease, paediatric, diagnosis, genomic medicine, South Africa, ICGNMD

## Abstract

Paediatric neuromuscular diseases are under-recognised and under-diagnosed in Africa, especially those of genetic origin. This may be attributable to various factors, inclusive of socioeconomic barriers, high burden of communicable and non-communicable diseases, resource constraints, lack of expertise in specialised fields and paucity of genetic testing facilities and biobanks in the African population, making access to and interpretation of results more challenging. As new treatments become available that are effective for specific sub-phenotypes, it is even more important to confirm a genetic diagnosis for affected children to be eligible for drug trials and potential treatments. This perspective article aims to create awareness of the major neuromuscular diseases clinically diagnosed in the South African paediatric populations, as well as the current challenges and possible solutions. With this in mind, we introduce a multi-centred research platform (ICGNMD), which aims to address the limited knowledge on NMD aetiology and to improve genetic diagnostic capacities in South African and other African populations.

## Introduction

Neuromuscular disease (NMD) includes a wide spectrum of rare diseases with primary abnormalities in the peripheral nervous system. Progressive muscle weakness is a key feature, as well as impaired ambulation, joint contractures, skeletal deformities, altered sensory perception and respiratory failure. NMDs are predominantly monogenic in origin, and affect at least 20 million children and adults globally, with prevalence rates estimated between 1 and 10 per 100,000 (1). These estimations do not include any of the African populations, which remain significantly understudied (2, 3).

Paediatric NMDs are historically associated with a poor prognosis, however, treatment strategies and options have rapidly expanded (4). This is illustrated by recent advances in therapies for Duchenne- and facioscapulohumeral muscular dystrophies (5, 6), and spinal muscular atrophy (7). It is important to identify the population specific genetic origin of NMDs in understudied populations (8), and to apply the knowledge in local new-born screening programs to identify patients who will benefit from specific treatment strategies (9).

The South African paediatric patients stem from a unique diversity of African, Asian and Caucasian populations. From data presented here it is evident that NMDs have unique phenotypes and genetic variants in these populations. The lack of data, restricted genetic services, along with socio-economic demands and national health priorities, limit the options and access to treatment for patients with NMDs. Here, we provide perspectives on NMD phenotypes recognised in the South African paediatric populations, clinical and molecular diagnostic challenges, and timely opportunities to address the lack of knowledge and capacity in the form of a multi-centre collaborative genomics medicine platform.

## Paediatric NMD in the South African populations

### Diagnostic odyssey

Similar to many African populations, South African patients with suspected NMDs, in both the private and public sector, experience a diagnostic odyssey. Only a small number of specialized clinics exist as the portal for disease diagnosis and translational medicine. Moreover, indigenous, or cultural aspects that result in lack of referral and/or referral bias should be acknowledged (8). Further barriers to diagnosis and referral are practitioners lacking training in NMDs. An effective diagnostic approach requires knowledge of phenotypes and genotypes (pathogenic variants and background genetics) in all local populations. Common and population specific NMD phenotypes have been identified, expressed on a genetic background for indigenous African populations that are relatively unreported in genomic databases, as summarised in Table 1 (10–15). Moreover, with Southern Africa being one of the global hot spots for both infectious and non-communicable acquired diseases, national health priorities and subsequent national support structures do not include or promote rare genetic diseases. This is evident in the delay of diagnosis and comprehensive management**,** including counselling and access to novel therapeutic interventions.

**Table 1:** Known pathogenic variants which are being screened for in patients with neuromuscular and/or mitochondrial or metabolic disease phenotypes in South African populations.

Pathogenic variants	RefSNP	Population allele frequency	Phenotype associated	References
NM_000540.3(*RYR1*): c.8342_8343delTA p.Ile2781ArgfsX49	–	n/a	Autosomal recessive centronuclear myopathy	(15)
NM_000540.3(*RYR1*): c.14524G > A p.Val4842Met^†^	rs193922879	n/a	Autosomal recessive centronuclear myopathy	(15)
NM_004453.4(*ETFDH*): c.1448C > T p.Pro483Leu	rs377656387	n/a	Multiple Acyl dehydrogenase deficiency	(13)
NM_004453.4(*ETFDH*): c.1067G > A p.Gly356Glu	–	n/a	Multiple Acyl dehydrogenase deficiency	(13)
NM_000344.4(*SMN1*): Exon 7 deletion	–	1 in 50 (Black)	Spinal muscular atrophy	(14, 16)
		1 in 23 (Caucasian)		
NM_005609.4(*PYGM*): c.148C > T p.Arg50ter	rs116987552	n/a	McArdle disease (Glycogen storage disease, type V)	
NM_002437.5(*MPV17*): c.106C > T p.Gln36Ter, Ser25Profs*49	rs754051090	1 in 68 (Black) (0.01471)	MPV17-related hepatocerebral mtDNA depletion syndrome	(11)
NM_004006.3(*DMD*): Exonic duplications and deletions, MLPA	–	n/a	Duchenne and Becker Muscular Dystrophy	
NM_024301.5(*FKRP*): c.1100T > C p.Ile367Thr	rs1555739020	1 in 100 (Afrikaner Caucasian)	FKRP-related muscular dystrophy	(17)
NM_024301.5(*FKRP*): c.826C > A p.Leu276Ile	rs28937900	n/a	FKRP-related muscular dystrophy	(17)

^†^ This variant is known to co-segregate in cis with the splice variant NM_00540.3(RYR1):c.10348-6C > G, which is not routinely tested for in this population; n/a, not available.

### Muscular dystrophies

Muscular dystrophies include Duchenne and Becker's muscular dystrophy, congenital muscular dystrophies with and without connective tissue involvement, limb-girdle muscular dystrophies and various other forms named according to the pattern of affected muscle groups. Duchenne muscular dystrophy (DMD) is the most common and severe of the inherited dystrophies, with a worldwide estimated incidence of 1 in 3,500 live male births (18). DMD manifests in boys between 2 and 5 years of age and presents with delayed motor milestones, associated features calf pseudohypertrophy, lumbar lordosis and weakness of the neck flexor muscles (19). A genetic service for DMD and BMD was initiated in Cape Town in 1987 and was the first offered nationally in the state health service (20). Overall minimum prevalence rates of 1/100,000 were calculated, a markedly low prevalence of DMD in the indigenous black population, 1/250,000, contributed to the overall low prevalence (20). Multiple ligase probe amplification (MLPA) was introduced by the National Health Laboratory Service (NHLS) in 2007 which allowed for identification of exonic rearrangements across the gene, not just the hotspots (21) and significantly improved pathogenic variant detection rate from 30 to 45% (19). As an alternate diagnostic aid, the role of cultured skin melanocytes was explored in South Africa (SA, 22).

Looking at other African populations, a study from Cameroon identified 17 boys with DMD, diagnosis was delayed into adolescence and significant under-recognition of DMD in the country was hypothesized (23). Patients with LGMD (*n* = 67) were reported from Mali with onset predominantly in the first decade but the group lacked access to genetic diagnostic closure (24). North African countries are more resourced for access to genetic testing, as illustrated by researchers from Morocco who identified six novel pathogenic variants in the DMD gene following whole dystrophin gene sequencing (25). The perception that novel variants are prevalent in Africa are supported by various case reports (17, 26–29).

Genetic testing for other forms of congenital muscular dystrophy is not routinely available through the state service in SA. Awareness of the novel FKRP-related muscular dystrophy founder mutation in South African Afrikaner populations has affected clinical practice through raised awareness and accordingly targeting diagnostic assessments (Table 1, 17). In such cases, an opportunistic muscle biopsy during other essential surgical interventions may be performed and the probable diagnosis may be supported by immunohistochemical stains on light microscopy as well as electron microscopy findings.

### Spinal muscular atrophy

Spinal muscular atrophy (SMA) is the second most common autosomal recessively inherited disorder caused by the homozygous loss of the *SMN1* gene (30). It is characterized by degeneration of alpha motor neurons in the spinal cord which results in progressive proximal muscle weakness (30). The incidence of SMA varies between 1 in 6,000 and 1 in 10,000 and carrier frequency between 1 in 40 to 1 in 60 globally (31). In SA, the incidence in the indigenous African populations is estimated to be 1 in 3,574 and in European ancestry populations 1 in 1,945, with carrier frequency of 1 in 50 and 1 in 23 in the Black African and European populations respectively (16). Lower expression of homozygous *SMN1* gene deletion is reported in Black South African populations who have phenotypes compatible with SMA. *SMN1* deletion heterozygosity was found in at least 70% of these patients (16). A proportion of these patients had additional clinical features inclusive of myopathic facies. The same findings were not replicated in the Western Cape region of SA, where the genotype and phenotype were concordant with international inclusion criteria for SMA (14). Larger national population studies are needed to understand the true prevalence of homozygous *SMN1* gene deletions in children with SMA phenotypes across South African ancestries, and to understand the sub-population with additional clinical features. Genetic testing is available through the public and private sectors and for a small proportion carrier testing can be undertaken (Table 1).

Newer therapies such as antisense oligonucleotides and gene therapies, whilst available and approved in some high-income countries, are precluded due to the cost in many low- and middle-income countries. Such settings are dependent on compassionate access programs or being part of research studies. Most patients with SMA are therefore left without access to treatment which raises many ethical and moral concerns. In line with this, neonatal screening has not been taken up in SA.

### Congenital myopathies

Congenital myopathies are a group of genetically inherited muscle disorders clinically characterized by hypotonia and weakness (32). Congenital myopathies were traditionally classified based on morphological features on muscle biopsy, however, this has evolved to a genetic classification based on the mutation(s) present in addition to the clinical phenotype and histological profile. The major groups of congenital myopathies include centronuclear myopathy (CNM), central core disease (CCD), nemaline myopathy, congenital fibre-type disproportion and myosin storage myopathy.

The *RYR1* gene is located on chromosome 19q13.1, contains 106 exons (33) and encodes *RYR1*, the principal sarcoplasmic reticulum calcium release channel that is involved in excitation-contraction coupling (34). Dominant variants in the *RYR1* gene result in CCD and malignant hyperthermia susceptibility (MHS) trait. Recessive variants in the *RYR1* gene result in multi-minicore disease (MMD), CNM and some cases of CCD. In 2010, 12 patients from SA were found to have variants in the *RYR1* gene from a cohort of 24 globally recruited patients with genetically unresolved CCD. The main genetic finding was compound heterozygosity for *RYR1* nonsense and missense variants. Two recurrent *RYR1* variants had associated common haplotypes suggesting a founder effect. Centronuclear myopathy is the most common form of congenital myopathy in the Western Cape province of SA, despite a lower prevalence in other populations (15). Locally, we are only able to perform genetic testing for the two founder variants in the *RYR1* gene (see Table 1). Other forms of congenital myopathies may be suspected based on the phenotypic features and findings on muscle biopsy. There is increasing recognition of expression of *STAC3* variants in Africa (12). This condition originally recognized as “Native American Myopathy” prevalent in the Lumbee community living for several generations in Carolina, USA, is now reported to be expressed in African populations, specifically NM_145064.3(STAC3):c.851G > C (p.Trp284Ser) (35, 36).

### Inherited neuropathies

CMT may affect the motor nerves, motor and sensory nerves with/without autonomic nerve and have variable involvement across the sub-groups in each category (37). There is marked diversity in the clinical presentation and genetic heterogeneity. Children present with more autosomal recessive forms of the disease due to higher prevalence of point mutations (38). The clinical phenotype typically consists of distal amyotrophy and weakness, usually associated with distal sensory impairment. These features may be accompanied by sensory ataxia, skeletal deformities, contractions and reduced or absent deep tendon reflexes (39). Supportive investigations including nerve biopsy and neurophysiological studies, is helpful to deeply phenotype patients with complex disease.

The global prevalence of hereditary motor and sensory neuropathies is 82.3 per 100,000. The most commonly inherited childhood form of CMT in Europe and North America, accounting for 40% of all cases of CMT, is the demyelinating form, CMT1A caused by a duplication of the *PMP22* gene (40). Limited epidemiological data is available for Africa. A community-based study from Egypt estimated CMT prevalence to be 12 per 100,000 (3, 41, 42).

A recent systematic review on CMT in Africa identified 29 reports, most from North Africa (*n* = 22) and few documented inclusion of paediatric age groups (*n* = 7) (43). Demyelinating forms were the most common with autosomal recessive inheritance pattern in 91.2% of families. CMT-associated variants were reported in 11 genes. The analysis noted disparity in the genes more commonly reported in European and North American populations, which appear to be less often expressed or identified in African populations, especially CMT1A. The commonest subtype of CMT in North Africa is CMT2B1 (LMNA), an autosomal recessive inheritance disease which is most likely attributable to the high rate of consanguinity in the region (44).

Currently the only genetic test offered by the NHLS in SA is screening for *PMP22* deletion/duplication.

### Mitochondrial disease and metabolic myopathies

Primary mitochondrial disease (PMD) refers to conditions associated with dysfunctional oxidative phosphorylation (OXPHOS), whereas metabolic myopathies encapsulate a large group of inborn errors of metabolism affecting energy metabolism resulting in muscle diseases (45). In PMD, any gene affecting the structural proteins of the OXPHOS system are involved (46). Up to 1,900 proteins are involved in the synthesis of ATP (47, 48). Most of these genes are encoded by nuclear DNA (nDNA), while a small subset of 13 essential subunits of the mitochondrial respiratory chain is encoded by mitochondrial genome (mtDNA).

Inherited defects in both mtDNA and nDNA encoded mitochondrial proteins can result in multisystemic phenotypes ranging from mild manifestations to severe myopathies and encephalopathies (49), with at least 338 genes (302 nDNA, 36 mtDNA) associated with primary MD (50).

Using a limited screening approach, primarily focusing on common mtDNA variants and a handful of common nDNA genes over a period of approximately 26 years, Meldau et al. (51) genetically confirmed primary mitochondrial disease in 9.6% of samples tested from a South African cohort. Although mtDNA findings from their cohort resembled data from other well-described populations, marked paucity of nDNA defects identified in common genes highlights the need for more extensive screening methods in such diverse, genetically understudied populations as found in sub-Saharan Africa (51). Even with the use of more extensive genetic panels, nDNA defects could not be identified in a handful of cases with biochemically proven disease, indicating that these panels may not include novel disease-gene associations yet to be identified in African cohorts (52).

In a study by Schoonen et al., it was observed that mtDNA variants are mostly absent in paediatric MD cohorts with confirmed muscle respiratory chain deficiencies from the northern provinces of SA, and a low diagnostic confirmation rate when using a nDNA panel-based next generation sequencing approach (12, 53).

For metabolic myopathies, more extensive genetic approaches have already proved valuable in identifying a unique nuclear *ETFDH* variant associated with secondary mitochondrial dysfunction in the Afrikaner population (Table 1, 13).

### Muscle channelopathies

Muscle channelopathies are rare inherited diseases due to variants in muscle ion channels, including chloride, sodium, calcium, and potassium channels. An increase or decrease in muscle membrane excitability occurs, manifesting a spectrum of related clinical disorders. Non-dystrophic myotonias have muscle stiffness and pain due to delayed relaxation after muscle contraction and periodic paralysis, characterized by episodes of flaccid paralysis. Autosomal dominant or sporadic inheritance is typical for most channelopathies, except for chloride channel myotonia which are recessively inherited. In SA there is limited access to testing for myotonic dystrophy in the public sector.

There is a paucity of data regarding muscle channelopathies in Africa. A population study from Egypt reported a lifetime prevalence of 54 per 100,000 (54, 55). A case report documented the first case of an African family (spanning five generations) with hypokalaemic periodic paralysis due to the *CACNA1S* R1239H variant. In this family, a later age of onset and specific triggering factors due to African conditions were noted (56). Another Dutch study explored the expression of Myotonic dystrophy type 2 (DM2). The group concluded that DM2 variants in Europe and North Africa originated from a single ancestral founder haplotype based on common expression found in both Dutch patients and a family of Moroccan ancestry (57).

### Congenital and childhood onset myasthenia's

Congenital myasthenic syndromes compose a group of neuromuscular transmission disorders due to variants in genes encoding proteins involved in the function of the motor end-plate (58) The condition is clinically characterised by abnormal fatiguability or weakness of extraocular, facial bulbar, truncal, respiratory or limb muscles (58) The diagnosis may be suspected based on the phenotype, supported by a decremental electromyographic response or abnormal single-fibre electromyographic response (59). A definitive genetic diagnosis is made with whole exome sequencing (59) and not routinely available locally.

The prevalence of CMS in Africa is unknown, however, a single founder mutation originating in North Africa is a frequent cause of congenital myasthenia in this region. A single truncation mutation (1293insG) in the acetylcholine receptor epsilon subunit gene, *CHRNE*, was most often identified in CMS families from North Africa (60). Present therapies for CMS include cholinergic agonists, long-lived open channel blockers of acetylcholine receptor ion channel and adrenergic agonists (59). Some drugs beneficial in one particular type of CMS, may be detrimental in another (59), underscoring the need for a clear genetic diagnosis.

Children with ocular myasthenia gravis (MG) from Asia are four times more common and children with African ancestry are 2–3-fold more frequent, than European children both in those of prepubertal and postpubertal ages at onset (61, 62). Genetic influences are suspected. Treatment-resistant ophthalmoplegia appears to be more frequent in African and Asian juvenile MG cohorts compared to Europeans (63, 64). Genetic and muscle gene expression studies point to dysregulated muscle atrophy signalling and mitochondrial metabolism pathways as pathogenetic mechanisms underpinning treatment-resistant ophthalmoplegia in susceptible individuals.

## Genomic medicine: challenges, opportunities, and prospects

Genomic medicine is a rapidly emerging medical discipline and can be defined as the use of genomic information about a person for their clinical care and management (e.g., for diagnostic or therapeutic decision-making). It also includes the health outcomes and policy implications of that clinical use. The discipline has several benefits at a population level, including identification of at-risk individuals before disease onset, compilation of population-specific genomic data, and the eventual implementation of precision medicine strategies.

As summarised in the previous sections, there are a limited number of genomic studies on NMD in the South African population, with no prevalence data. This is a significant shortcoming since it is widely acknowledged that sub-Saharan African populations are particularly pertinent for genomic medicine as they harbour the greatest genetic diversity globally (65, 66). They are also important for studying gene-environment interactions since the African continent has many diverse environments and climatic conditions to which its inhabitants have adapted over millions of years (65, 67). Unfortunately, despite these obvious benefits and opportunities, there are currently several *barriers* to the implementation of genomic medicine for NMD in SA and elsewhere in Africa, including:

1. Lack of government support for formalised bioinformatics training especially for clinicians.
2. Resource constraints in state genetic diagnostic laboratories.
3. Limited genomic data publicly available, with specific challenges to predict pathogenic variants in African populations (68).
4. A legacy of “helicopter science” where researchers obtain biospecimens locally and ship these to overseas laboratories with no benefit to the study participants or to local researchers (69).

All of these obstacles need to be addressed urgently, however, before embarking on genomic medicine strategies, there are several ethical issues that need to be considered. Historically, African populations have been exploited and abused, particularly in the context of genetic studies (55). Issues such as appropriate consenting practices (informed, tiered consent is ideal as this has a greater chance for *protection of autonomy, dignity and most importantly, informed choice for individuals in every aspect of the use of their data and samples*) (70, 71) and the unregulated use of genomic data, need to be considered*.* Recently, at the World Conference on Research Integrity, the Cape Town Declaration on Fair Research Partnerships was launched which aims to deal with “helicopter research” and related ethical and moral issues (69). Additionally, the Summer internship for INdigenous peoples in Genomics (SING) Consortium has designed an “ethical framework” to increase inclusion of diverse groups in genomic research (72). Purposely excluding vulnerable populations from genetic studies would prevent them from benefiting from genomic medicine strategies.

**Figure 1:**
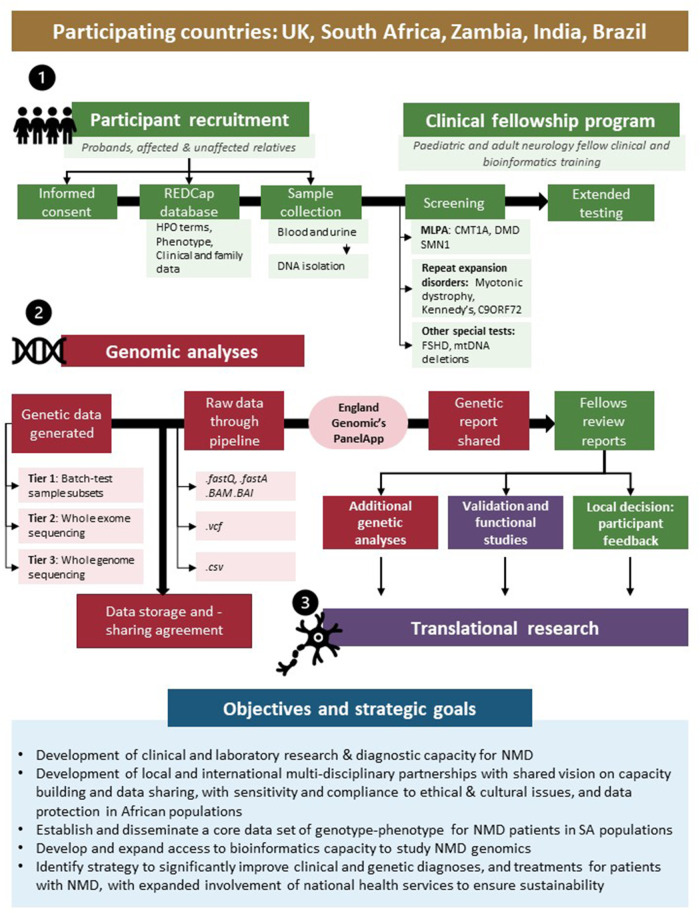
International Centre for Genomic Medicine in Neuromuscular Disease (ICGNMD) research strategy and SA-specific outcomes. The study, involving four South African academic centres as part of a six-nation global NMD genomic study, is structured in three phases: a clinical fellow training- (**1**), genomic- (**2**), and translational phase (**3**). Green indicates local clinical fellow involvement; red indicates collaborative genomics strategy and purple specific collaborative follow-up studies where novel findings are investigated.

Taking many of these challenges and opportunities into account, the International Centre for Genomic Medicine in Neuromuscular Disease (ICGNMD, www.ucl.ac.uk/icgnmd) was launched in 2019. It consists of six participating countries with a structure and specific objectives as summarised in Figure 1. Four SA academic centres participate in this endeavour with recognition of an expanding network approach to address these core challenges of studying NMD in Southern African populations, and to contribute to the ICGNMD's ultimate aims of growing international knowledge of the global genetic architecture of inherited NMDs, to enable improved diagnostic and therapeutic outcomes for patients.

## Conclusions

Closing the diagnostic gap in the South African paediatric population with neuromuscular diseases could not be more pivotal at this time. Overcoming challenges, with the support of various role players, would be imperative to achieve this. Through collaborations, such as with the ICGNMD study, we hope to significantly contribute to this process. The ultimate goal would be to build locally sustainable models for use in LMIC, including many African populations, however this would require the continuous backing from all relevant stakeholders.

## Data Availability

The original contributions presented in the study are included in the article/Supplementary Material, further inquiries can be directed to the corresponding author/s.
